# Oral supplementation of vitamin D is safe and can be an effective strategy to fill the nutritional gap

**DOI:** 10.1017/jns.2023.1

**Published:** 2023-03-09

**Authors:** Jutta Dierkes, Manfred Eggersdorfer

**Affiliations:** 1 Centre for Nutrition, Department of Clinical Medicine, University Bergen, Bergen, Norway; 2 Department of Internal Medicine, University Medical Center Groningen, Groningen, The Netherlands

Dear Editor,

This is with reference to the article ‘Physiological significance of vitamin D produced in skin compared with oral vitamin D’ published in *Journal of Nutritional Science* (2022), vol. 11, e13, page 1–5. We would be grateful if the *Journal of Nutritional Science* could publish this letter.

A recent paper by Fraser^(1)^ from the Sydney School of Veterinary Science, Faculty of Science, The University of Sydney addresses the safety of vitamin D applications describing an experiment in squirrel monkeys which has – in his view – shown that long-term supply of oral vitamin D in non-toxic amounts causes atherosclerosis in large arteries. In this paper, the author speculates about an aspect of vitamin D physiology that has been ignored – the mechanisms for the transport and processing from the two sources of vitamin D either from food or by endogenous synthesis in skin. He argues that the transport mechanism for the two sources is quite different. In the light of the angiotoxicity caused by oral vitamin D in animal experiments, he proposes that alternative strategies to food fortification and/or supplementation for addressing widespread vitamin D deficiency in humans should be considered.

The paper is a perspective paper, drawing on mechanistic insights, opinion and speculation to support physiological observations. The author deviates from typical intakes, recommendations and available clinical evidence for vitamin D. The author confuses nutritional and toxicological levels of vitamin D and 25(OH)D and contrary to authoritative health guidance, proposes UV-exposure and transdermal administration as preferred routes for obtaining optimal vitamin D status. While in nutritional studies, the effect of a bioactive is studied with doses in the range of a safe daily intake toxicological studies aim to identify the dose at which adverse effects happen. He argues that animal studies demonstrating adverse outcomes for vascular pathology underpin his argumentation. However, the primary *in vivo* study cited in the paper is one squirrel monkey trial – in this trial monkeys were fed a high dose of vitamin D, equivalent to 1000 μg/d for humans. This dose exceeds the established Upper Tolerable Level recommendation either provided by the Institute of Medicine (IOM) or the European Food and Safety Authority (EFSA) by 10-fold and exceeds the daily Dietary Reference Intake (15 μg/d) by the factor 67^(2,3)^. In other words, the cited study is not a nutritional study.

The author speculates that oral intake of vitamin D is excessive beyond that which our body has evolved and the persistent oral consumption of 250 μg/d vitamin D is resulting in hypercalcaemia. However, the typical average oral intake of vitamin D from both food and supplements combined is in reality below 20 μg/d for people in Europe, in the USA and other regions^(4–8)^, and thus not comparable with the mentioned amount. This average intake is far below the IOM Upper Tolerable Level of 100 μg/d, while the author's level of concern of 250 μg/d is far above the Upper Tolerable Level. Reports of vitamin D overdose are rare in the literature. Several research groups and authorities evaluated the intake of vitamin D up to 15 000 IU/d (equivalent to 375 μg/d) and serum 25(OH)D up to 300 nmol/l were found to be safe^(9,10)^. A recent review suggested that hypercalcaemia may be caused by extremely high vitamin D intake and being responsible for arterial calcification. However, evidence to explain the mechanisms of action by which excess exogenous vitamin D promotes calcification is lacking^(11)^. In addition, there are also endogenous causes of hypervitaminosis D, such as increased production of 1,25(OH)2D as part of granulomatous disorders or lymphomas^(12)^.

In recent years, several randomised controlled clinical trials using amounts of vitamin D in the range of 2000–4000 IU/d (50 to 100 μg/d) for several years have been published. These trials are a valuable source of data on the safety of long-term use of recommended as well as for higher doses of vitamin D in adults. Even though this supplementation with vitamin D did not result in all trials in the risk reduction of major non-communicable diseases (NCDs), side effects and adverse events, on the other hand, were rare in these studies and not different from the placebo groups.

It is well accepted that blood 25(OH)D levels are the appropriate measure of status, not bound *v*. unbound vitamin D-binding protein. It is important to realise that the author states that 50–80 nmol/l 25(OH)D in blood represents a ‘good’ vitamin D status. This range is consistent with expert recommendations for adequate status (either 50 or 75 nmol/l, depending on expert group)^(4,13)^. Interestingly, this concentration is usually achieved in the majority of adults (in the absence of endogenous synthesis) by doses now recommended by authorities and nutrition societies as daily allowance of 15–20 μg/d^(14)^.

The author ignores the many studies and papers which describe the value and safety of food fortification and supplementation^(15)^. Food fortification, oral supplementation with vitamin D and/or moderate UV-exposure to stimulate vitamin D production in skin are rated as safe to improve the vitamin D status of the population. Considering the high prevalence of vitamin D deficiency worldwide and the resulting severe consequences for human health, experts recommend that vitamin D deficiency urgently needs to be addressed in many countries by governments and healthcare authorities^(5,6,15–17)^.

The author would prefer vitamin D to be obtained via synthesis in the skin. Modelling studies have demonstrated that it would be challenging for many people to reach optimal D levels from sun exposure alone, based on seasonality, latitude, differences in skin type and cautions about sun exposure from dermatologists with the aim to prevent skin cancer. Reliance on endogenous synthesis does not work in many countries in the northern and southern hemisphere. For example, countries like Canada, Germany, the UK, Denmark, Norway, Finland, just to mention a few, do not get the sun with a wavelength range of 290–320 nm required for the endogenic synthesis for 5–8 months during the year^(18)^. Additionally, ability to produce vitamin D in the skin declines with aging^(19)^. For this reason, the oral intake via food, fortified foods and/or supplements is an opportunity to fill the gap and achieve the recommended intake. The argument that oral supply of vitamin D has only been a strategy for the last 100 years is not right. Supplementation using cod liver oil tran is established for several hundreds of years and demonstrated to be safe and effective. Also, his proposal for transdermal injections of vitamin D can be a choice for patients but it is not practical for larger population groups.

The author explains his theory in detail how oral vitamin D is detrimental for vascular physiology, however, a search of the clinical literature provides plenty of evidence that vitamin D supplementation supports cardiovascular health and provides a wide range of other health benefits^(10,20–22)^. For example, observational studies have associated vitamin D supplementation with lower risk for heart disease, and neutral effect on coronary artery calcified plaque burden. In RCTs, vitamin D supplementation is protective or neutral for blood pressure^(20)^. So, based on many human studies, vitamin D fortified foods and supplements in the recommended doses are a safe and can be an effective strategy to fill the nutritional gap.

It is also noteworthy, that Fraser is not discussing at all the differences of vitamin D2 and D3. Endogenously synthesised vitamin D will be vitamin D3, while orally obtained vitamin D can be either D2 or D3. There is some evidence that these forms have different effects on vitamin D status and metabolism^(23,24)^, and it was also suggested that the prevention of, for example, falls was different for supplements with vitamin D2 and D3^(25)^. Differences between vitamin D2 and D3 may lead to confusion concerning vitamin D supplementation.

While there are clearly opportunities for more research on the role of vitamin D supplementation and the physiological significance of vitamin D produced in skin *v*. oral supplementation in our opinion, the author is overstating the significance of his findings. We do not agree the findings presented in this paper should form the basis for vitamin D supplementation in humans. The findings in this paper are far from conclusive and should be placed in the proper context because the conclusion in the paper ‘Physiological significance of vitamin D produced in skin compared with oral vitamin D’ is misleading and is not supported by the interesting, but very limited findings of this paper.
